# Spontaneous resolution and crystal structure of (2*S*)-2-(3-nitro­phen­yl)-3-phenyl-2,3,5,6-tetra­hydro-4*H*-1,3-thia­zin-4-one; crystal structure of *rac*-2-(4-nitro­phen­yl)-3-phenyl-2,3,5,6-tetra­hydro-4*H*-1,3-thia­zin-4-one

**DOI:** 10.1107/S2056989018003444

**Published:** 2018-03-06

**Authors:** Hemant P. Yennawar, Heather G. Bradley, Kristen C. Perhonitch, Haley E. Reppert, Lee J. Silverberg

**Affiliations:** a The Pennsylvania State University, Dept. Biochemistry and Molecular Biology, University Park, Pa 16802, USA; b Pennsylvania State University, Schuylkill Campus, 200 University Drive, Schuylkill Haven, PA 17972, USA

**Keywords:** crystal structure, nitro group, 1,3-thia­zin-4-one, spontaneous resolution

## Abstract

The crystal structures of isomeric *rac*-2-(4-nitro­phen­yl)-3-phenyl-2,3,5,6-tetra­hydro-4*H*-1,3-thia­zin-4-one (C_16_H_14_N_2_O_3_S) (**1**) and (2*S*)-2-(3-nitro­phen­yl)-3-phenyl-2,3,5,6-tetra­hydro-4*H*-1,3-thia­zin-4-one (C_16_H_14_N_2_O_3_S) (**2**) are reported here. While **1** crystallizes in a centrosymmetric space group, the crystal of **2** chosen for data collection has mol­ecules only with (2*S*) chirality. This is the result of spontaneous resolution during crystallization, as the synthesis produces a racemic mixture. A crystal with (2*R*) mol­ecules was also found in the same crystallization vial (structure factors available). The six-membered thia­zine ring in both **1** and **2** displays an envelope conformation with the S atom forming the flap. The aryl rings in both structures adopt an approximate V shape with angles between their planes of 46.97 (14)° in **1** and 58.37 (10)° in **2**. In both structures, the mol­ecules form layers in the *ab* plane. Within such a layer in **1**, one of the O atoms of the nitro­phenyl group accepts a C—H⋯O hydrogen bond from the CH group at position 5 of the thia­zine ring of a mol­ecule of opposite chirality, forming chains along the *a*-axis direction. Each of the thia­zine rings also participate in C—H⋯O bonds with the same carbon atom as above, resulting in chains along the *b*-axis direction, albeit of monochiral type. Adjacent layers are consolidated along the *c*-axis direction by pairs of parallel hydrogen bonds (C—H⋯O type) between the nitro­phenyl groups of enanti­omers. In **2**, the two C—H⋯O hydrogen bonds contribute to chain formation along the *b*-axis direction. Weak edge-to-face inter­actions between the aryl groups of neighbouring mol­ecules in **1**, and C—H⋯π inter­actions between a thia­zine ring CH group and a phenyl group of a neighboring mol­ecule in **2** are also observed.

## Abstract 2

One of the racemic title isomers crystallized in a centrosymmetric space group and the other spontaneously resolved into enanti­omers.

## Chemical context   

Compounds with an *N*-aryl-2,3,5,6-tetra­hydro-4*H*-1,3-thia­zin-4-one scaffold have been shown to have a wide variety of bioactivities, including anti­fungal (Qu *et al.*, 2013 ▸; Dandia *et al.*, 2004 ▸; Krumkains, 1984 ▸), anti­tubercular (Dandia *et al.*, 2004 ▸), anti­tumor (Chen *et al.*, 2012 ▸), anti­diabetic (Arya *et al.*, 2012 ▸), regulation of plant growth (Krumkains, 1984 ▸), cleavage of DNA (possible anti­tumor) (Dandia *et al.*, 2013 ▸), inhibition of cannabinoid receptor 1 (CB1) (Choi *et al.*, 2008 ▸), and inhibition of angiogenesis (possible treatment of eye disease, neoplasm, arteriosclerosis, arthritis, psoriasis, diabetes, and mellitus) (Chen *et al.*, 2012 ▸).

The spontaneous resolution of a racemic solution by direct crystallization to form a conglomerate, a mechanical mixture of separate homochiral crystals, is an uncommon but well-known phenomenon, recognized first by Pasteur in 1848 (Pasteur, 1848 ▸; Jacques *et al.*, 1981 ▸; Eliel & Wilen, 1994 ▸; Pérez-Garcia & Amabilino, 2007 ▸). It has even been used in the production of chiral active pharmaceutical ingredients (Bredikhin & Bredikhina, 2017 ▸). However, the reasons why this occurs with a minority of mol­ecules are not well understood (Pérez-Garcia & Amabilino, 2007 ▸) and have not yet yielded to attempts to predict occurrence (D’Oria, Karanertzanis & Price, 2010 ▸; Pérez-Garcia & Amabilino, 2007 ▸).
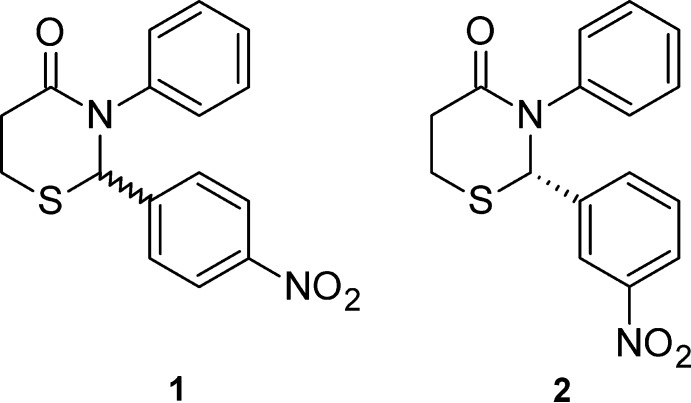


In this work, we report the spontaneous resolution and crystal structure of (2*S*)-2-(3-nitro­phen­yl)-3-phenyl-2,3,5,6-tetra­hydro-4*H*-1,3-thia­zin-4-one, **2**. We later collected another crystal from the vial and confirmed that it had the (2*R*) configuration (identical packing, structure factors available upon request). We also report the racemic (centrosymmetric) structure of the isomeric 2-(4-nitro­phen­yl)-3-phenyl-2,3,5,6-tetra­hydro-4*H*-1,3-thia­zin-4-one, **1**. We have previously reported the crystal structure of *rac*-2,3-diphenyl-2,3,5,6-tetra­hydro-4*H*-1,3-thia­zin-4-one (Yennawar & Silverberg, 2014 ▸).

## Structural commentary   

**Figure 1:**
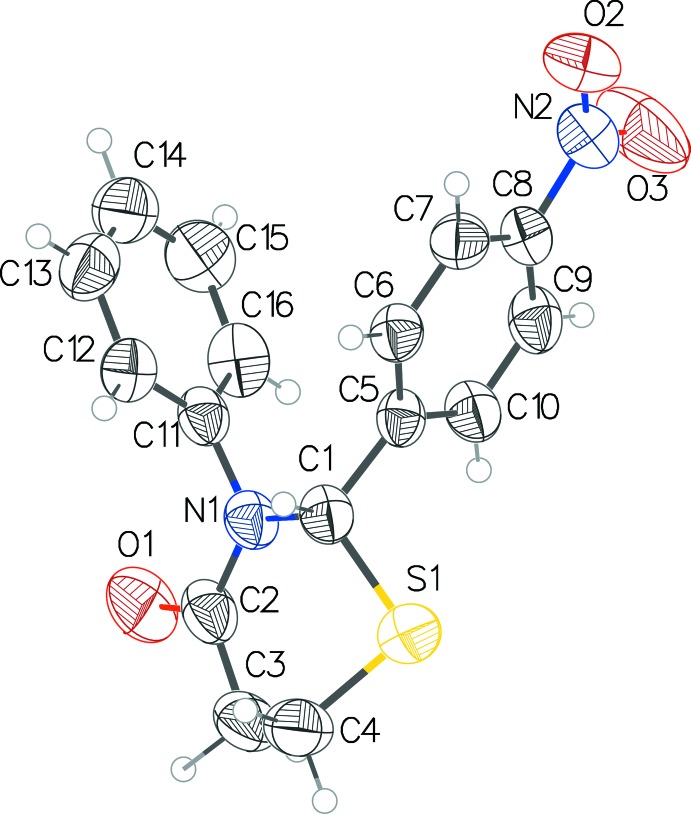
The mol­ecular structure of **1**, with displacement ellipsoids drawn at the 50% probability level.

Both structures **1** and **2** (Figs. 1 ▸ and 2 ▸) exhibit an envelope pucker conformation of the thia­zine ring with the sulfur atom forming the flap. The Cremer & Pople (1975) puckering parameters in **1** are: *Q* = 0.638 (3) Å, θ = 47.0 (3)°, φ = 339.8 (4)° and in **2**: *Q* = 0.6654 (16) Å, θ = 44.20 (17)°, φ = 353.8 (3)°. The aryl rings in both structures form an approximate V shape with inter-centroid distances of 3.964 (2) and 4.160 (2) Å, and inter­planar angles of 46.97 (14) and 58.37 (10)°, in **1** and **2**, respectively.

## Supra­molecular features   

**Figure 3:**
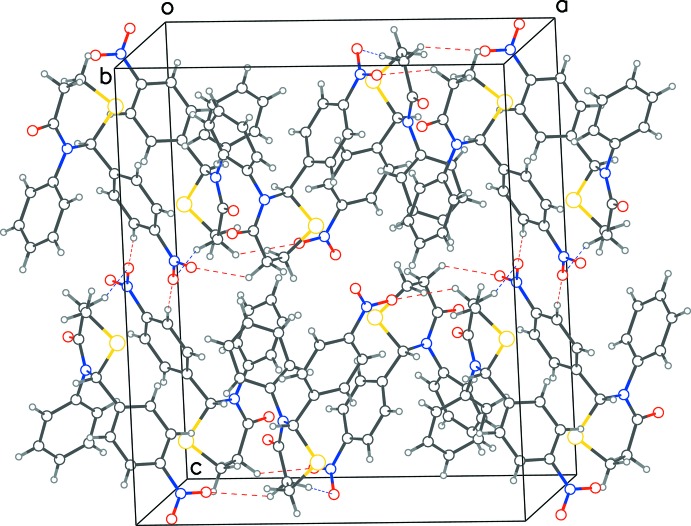
Packing diagram for **1**, showing the layering of mol­ecules in the *ab* plane. Red dotted lines show hydrogen bonds between enanti­omers and blue dotted lines show inter­actions between mol­ecules of same chirality.

In both structures, C—H⋯O inter­actions are observed (Tables 1 ▸ and 2 ▸, Figs. 3 ▸ and 4 ▸), resulting in layering of mol­ecules in planes parallel to (001). In each layer of structure **1**, one of the oxygen atoms of the nitro­phenyl group accepts a C—H⋯O hydrogen bond from the CH group at position 5 of the thia­zine ring of a mol­ecule of opposite chirality. This results in infinite chains of mixed chirality along the *a*-axis direction. The second oxygen atom of the nitro­phenyl group also accepts a hydrogen bond from the thia­zine 5-carbon atom, resulting this time in monochiral chains along the *b*-axis direction. Further, the stacking of layers along the *c*-axis direction is consolidated by pairs of parallel hydrogen bonds between the nitro­phenyl groups of enanti­omers. In **2**, a monochiral structure, the C—H⋯O hydrogen bonds between the chiral carbon atom and the 4-oxygen atom on the neighboring thia­zine ring results in a chain along the *b*-axis direction. The second hydrogen bond loops back to the second mol­ecule in the reverse direction of the same chain. While weak edge-to-face inter­actions [*Cg*⋯*Cg* distance of 5.340 (3) Å and an inter­planar angle of 84.99 (2)°] between the aryl groups of neighboring mol­ecules is observed in **1**, in **2**, the 6-carbon atom of the thia­zine ring inter­acts with the phenyl group in a C—H⋯π type inter­action [C4⋯*Cg* = 3.581 (2) Å].

## Database survey   

No substanti­ally similar crystal structures were found other than certain ones we have published, including 2,3-diphenyl-2,3,5,6-tetra­hydro-4*H*-1,3-thia­zin-4-one (Yennawar & Silverberg, 2014 ▸, 2015 ▸), 2-(3-nitro­phen­yl)-3-phenyl-2,3-di­hydro-4*H*-1,3-benzo­thia­zin-4-one (Yennawar *et al.*, 2013 ▸), and 2-(4-nitro­phen­yl)-3-phenyl-2,3-di­hydro-4*H*-1,3-benzo­thia­zin-4-one (Yennawar *et al.*, 2015 ▸).

## Synthesis and crystallization   

General: A two-necked 25 ml round-bottom flask was oven-dried, cooled under N_2_, and charged with a stir bar and the imine (6 mmol). 3-Mercaptopropionic acid (0.52 ml, 6 mmol) and then 2-methyl­tetra­hydro­furan (2.3 ml) were added and the solution was stirred. Pyridine (1.95 ml, 24 mmol) and finally, 2,4,6-tripropyl-1,3,5,2,4,6-trioxatri­phospho­rinane-2,4,6-trioxide (T3P) in 2-methyl­tetra­hydro­furan (50 weight percent; 7.3 ml, 12 mmol) were added. The reaction was stirred at room temperature and followed by TLC. The mixture was poured into a separatory funnel with di­chloro­methane and distilled water. The layers were separated and the aqueous was then extracted twice with di­chloro­methane. The organics were combined and washed with saturated sodium bicarbonate and then saturated sodium chloride. The organic was dried over sodium sulfate and concentrated under vacuum to give crude product.

2-(4-Nitro­phen­yl)-3-phenyl-2,3,5,6-tetra­hydro-4*H*-1,3-thia­zin-4-one (**1**): the crude product was recrystallized from 2-propanol to give a white powder. Yield: 1.397 g (74%). m.p. 410–412 K. Colorless blocks for data collection were grown by slow evaporation from 2-propanol solution.

2-(3-Nitro­phen­yl)-3-phenyl-2,3,5,6-tetra­hydro-4*H*-1,3-thia­zin-4-one (**2**): The crude product was recrystallized from 2-propanol to give a yellow powder. Yield: 1.121 g (59%). m.p. 415 K. Colorless blocks were grown by slow evaporation from ethanol solution; the (2*S*) and (2*R*) crystals had identical morphology. The stereochemical configuration of individual crystals was identified by solving the crystal structure. After several were found to be (2*S*), a crystal was found that was (2*R*).

## Refinement   

Crystal data, data collection and structure refinement details for both structures **1** and **2** are summarized in Table 3 ▸. The H atoms were placed geometrically and allowed to ride on their parent C atoms during refinement, with C—H distances of 0.93 Å (aromatic), 0.97 Å (methyl­ene) and 0.98 (meth­yl) and with *U*_iso_(H) = 1.2*U*_eq_(aromatic or methyl­ene C) or 1.5*U*_eq_(methyl C). In structure **2**, the absolute configuration for the chiral centres in the mol­ecule was determined as (2*S*) with a Flack absolute structure parameter of 0.09 (7) for 4055 Friedel pairs.

## Supplementary Material

Crystal structure: contains datablock(s) 1, 2. DOI: 10.1107/S2056989018003444/hb7733sup1.cif

Structure factors: contains datablock(s) 1. DOI: 10.1107/S2056989018003444/hb77331sup2.hkl

Supporting information file. DOI: 10.1107/S2056989018003444/hb77331sup4.mol

Structure factors: contains datablock(s) 2. DOI: 10.1107/S2056989018003444/hb77332sup3.hkl

Supporting information file. DOI: 10.1107/S2056989018003444/hb77332sup5.mol

Supporting information file. DOI: 10.1107/S2056989018003444/hb77331sup6.cml

Supporting information file. DOI: 10.1107/S2056989018003444/hb77332sup7.cml

CCDC references: 1826377, 1826376

Additional supporting information:  crystallographic information; 3D view; checkCIF report

## Figures and Tables

**Figure 2:**
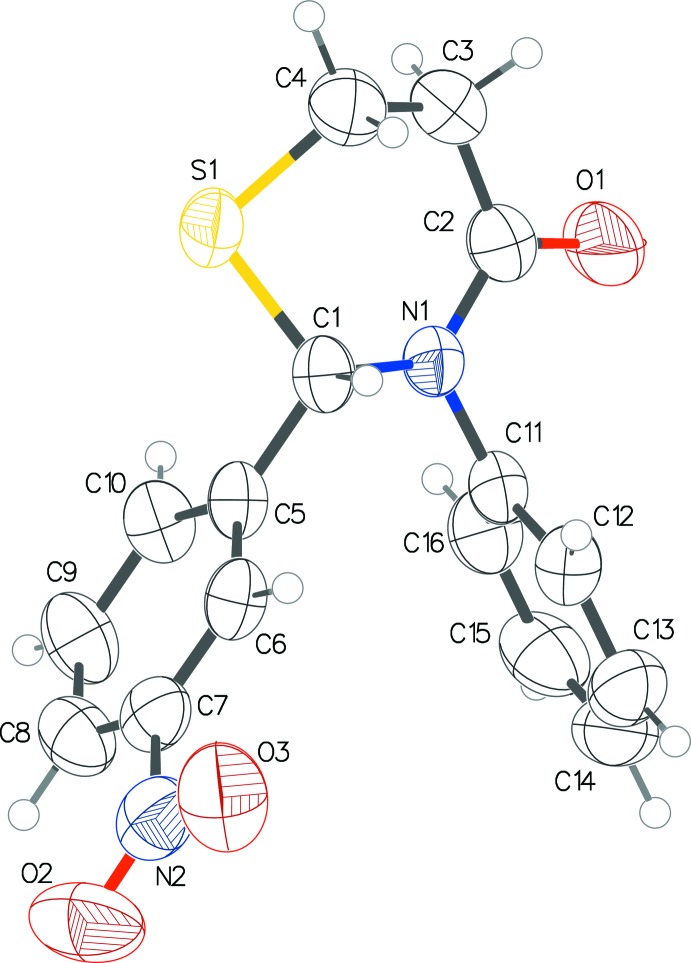
The mol­ecular structure of **2**, with displacement ellipsoids drawn at the 50% probability level.

**Figure 4:**
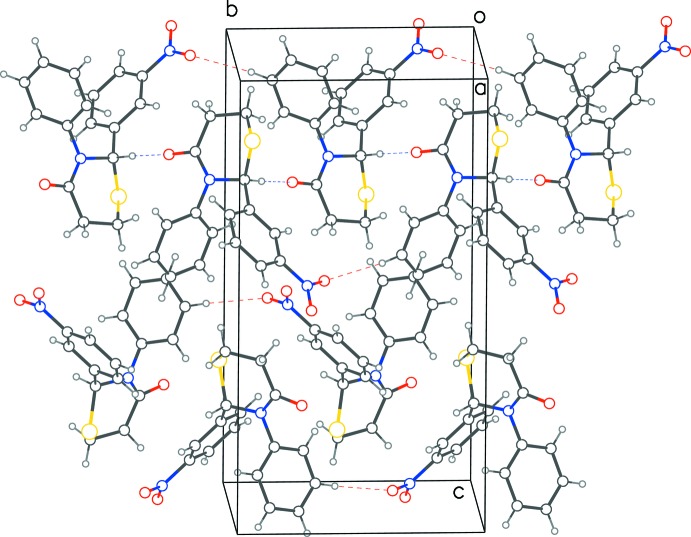
Packing diagram for **2**, showing the layering of mol­ecules in the *ab* plane. Blue dotted lines show hydrogen bonds between mol­ecules forming a chain in the *b*-axis direction and red dotted lines show a loop-back inter­action within each chain.

**Table 1:** Hydrogen-bond geometry (Å, °) for **1** [Chem scheme1]

*D*—H⋯*A*	*D*—H	H⋯*A*	*D*⋯*A*	*D*—H⋯*A*
C3—H3*A*⋯O2^i^	0.97	2.62	3.405 (4)	139
C3—H3*B*⋯O3^ii^	0.97	2.57	3.253 (5)	128
C7—H7⋯O2^iii^	0.93	2.50	3.417 (4)	170

Symmetry codes: (i) 
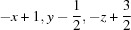
; (ii) 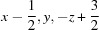; (iii) 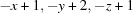.

**Table 2:** Hydrogen-bond geometry (Å, °) for **2** [Chem scheme1]

*D*—H⋯*A*	*D*—H	H⋯*A*	*D*⋯*A*	*D*—H⋯*A*
C1—H1⋯O1^i^	0.98	2.19	3.158 (2)	170
C15—H15⋯O3^ii^	0.93	2.58	3.501 (3)	174

Symmetry codes: (i) 
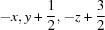
; (ii) 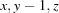.

**Table 3:** Experimental details

	**1**	**2**
Crystal data		
Chemical formula	C_16_H_14_N_2_O_3_S	C_16_H_14_N_2_O_3_S
*M*_r_	314.35	314.35
Crystal system, space group	Orthorhombic, *P* *b* *c* *a*	Orthorhombic, *P*2_1_2_1_2_1_
Temperature (K)	298	298
*a*, *b*, *c* (Å)	15.801 (6), 10.280 (4), 18.460 (7)	8.6877 (17), 9.6547 (19), 18.137 (4)
*V* (Å^3^)	2998.4 (19)	1521.3 (5)
*Z*	8	4
Radiation type	Mo *K*α	Mo *K*α
μ (mm^−1^)	0.23	0.23
Crystal size (mm)	0.2 × 0.16 × 0.09	0.21 × 0.19 × 0.18

Data collection		
Diffractometer	Bruker SMART CCD area detector	Bruker SMART CCD area detector
Absorption correction	Multi-scan (*SADABS*; Bruker, 2001 ▸)	Multi-scan (*SADABS*; Bruker, 2001 ▸)
*T*_min_, *T*_max_	0.154, 0.9	0.341, 0.9
No. of measured, independent and observed [*I* > 2σ(*I*)] reflections	26571, 3769, 2297	14176, 3775, 3144
*R*_int_	0.057	0.035
(sin θ/λ)_max_ (Å^−1^)	0.670	0.667

Refinement		
*R*[*F* ^2^ > 2σ(*F* ^2^)], *wR*(*F* ^2^), *S*	0.078, 0.216, 1.19	0.045, 0.121, 1.01
No. of reflections	3769	3775
No. of parameters	199	199
H-atom treatment	H-atom parameters constrained	H-atom parameters constrained
Δρ_max_, Δρ_min_ (e Å^−3^)	0.24, −0.48	0.32, −0.16
Absolute structure	–	Flack (1983 ▸), 4055 Friedel pairs
Absolute structure parameter	–	0.09 (7)

Computer programs: *SMART* and *SAINT* (Bruker, 2016 ▸), *olex2.solve* (Bourhis *et al.*, 2015 ▸), *SHELXS97* and *SHELXL97* (Sheldrick, 2008 ▸) and *OLEX2* (Dolomanov *et al.*, 2009 ▸).

## supplementary crystallographic information

### (2S)-2-(3-Nitrophenyl)-3-phenyl-3,4,5,6-tetrahydro-2H-1,3-thiazin-4-one (1) Crystal data

**Table:**

C_16_H_14_N_2_O_3_S	*D*_x_ = 1.393 Mg m^−^^3^
*M**_r_* = 314.35	Mo *K*α radiation, λ = 0.71073 Å
Orthorhombic, *P**b**c**a*	Cell parameters from 3516 reflections
*a* = 15.801 (6) Å	θ = 2.6–27.5°
*b* = 10.280 (4) Å	µ = 0.23 mm^−^^1^
*c* = 18.460 (7) Å	*T* = 298 K
*V* = 2998.4 (19) Å^3^	Block, colorless
*Z* = 8	0.2 × 0.16 × 0.09 mm
*F*(000) = 1312	

### (2S)-2-(3-Nitrophenyl)-3-phenyl-3,4,5,6-tetrahydro-2H-1,3-thiazin-4-one (1) Data collection

**Table:**

Bruker SMART CCD area detector diffractometer	3769 independent reflections
Radiation source: fine-focus sealed tube	2297 reflections with *I* > 2σ(*I*)
Graphite monochromator	*R*_int_ = 0.057
phi and ω scans	θ_max_ = 28.5°, θ_min_ = 2.2°
Absorption correction: multi-scan (SADABS; Bruker, 2001)	*h* = −20→21
*T*_min_ = 0.154, *T*_max_ = 0.9	*k* = −13→13
26571 measured reflections	*l* = −24→23

### (2S)-2-(3-Nitrophenyl)-3-phenyl-3,4,5,6-tetrahydro-2H-1,3-thiazin-4-one (1) Refinement

**Table:**

Refinement on *F*^2^	Primary atom site location: iterative
Least-squares matrix: full	Secondary atom site location: difference Fourier map
*R*[*F*^2^ > 2σ(*F*^2^)] = 0.078	Hydrogen site location: inferred from neighbouring sites
*wR*(*F*^2^) = 0.216	H-atom parameters constrained
*S* = 1.19	*w* = 1/[σ^2^(*F*_o_^2^) + (0.1*P*)^2^] where *P* = (*F*_o_^2^ + 2*F*_c_^2^)/3
3769 reflections	(Δ/σ)_max_ < 0.001
199 parameters	Δρ_max_ = 0.24 e Å^−^^3^
0 restraints	Δρ_min_ = −0.48 e Å^−^^3^

### (2S)-2-(3-Nitrophenyl)-3-phenyl-3,4,5,6-tetrahydro-2H-1,3-thiazin-4-one (1) Special details

**Table:**

Experimental. The data collection nominally covered a full sphere of reciprocal space by a combination of 4 sets of ω scans each set at different φ and/or 2θ angles and each scan (10 s exposure) covering -0.300° degrees in ω. The crystal to detector distance was 5.82 cm.
Geometry. All esds (except the esd in the dihedral angle between two l.s. planes) are estimated using the full covariance matrix. The cell esds are taken into account individually in the estimation of esds in distances, angles and torsion angles; correlations between esds in cell parameters are only used when they are defined by crystal symmetry. An approximate (isotropic) treatment of cell esds is used for estimating esds involving l.s. planes.
Refinement. Refinement of F^2^ against ALL reflections. The weighted R-factor wR and goodness of fit S are based on F^2^, conventional R-factors R are based on F, with F set to zero for negative F^2^. The threshold expression of F^2^ > 2sigma(F^2^) is used only for calculating R-factors(gt) etc. and is not relevant to the choice of reflections for refinement. R-factors based on F^2^ are statistically about twice as large as those based on F, and R- factors based on ALL data will be even larger.

### (2S)-2-(3-Nitrophenyl)-3-phenyl-3,4,5,6-tetrahydro-2H-1,3-thiazin-4-one (1) Fractional atomic coordinates and isotropic or equivalent isotropic displacement parameters (Å^2^)

**Table:**

	*x*	*y*	*z*	*U*_iso_*/*U*_eq_	
C1	0.38874 (16)	0.8032 (2)	0.80475 (15)	0.0493 (7)	
H1	0.3458	0.8713	0.7995	0.059*	
C2	0.32207 (18)	0.6103 (3)	0.86948 (17)	0.0613 (8)	
C3	0.3466 (2)	0.6614 (3)	0.94234 (17)	0.0744 (10)	
H3A	0.3941	0.6104	0.9597	0.089*	
H3B	0.2998	0.6462	0.9752	0.089*	
C4	0.3704 (2)	0.8027 (4)	0.94716 (18)	0.0746 (10)	
H4A	0.3901	0.8224	0.9957	0.090*	
H4B	0.3212	0.8562	0.9373	0.090*	
C5	0.44572 (15)	0.8058 (2)	0.73915 (15)	0.0446 (6)	
C6	0.42995 (16)	0.8917 (2)	0.68348 (15)	0.0482 (7)	
H6	0.3860	0.9513	0.6880	0.058*	
C7	0.47813 (18)	0.8909 (3)	0.62117 (15)	0.0551 (7)	
H7	0.4674	0.9495	0.5839	0.066*	
C8	0.54241 (16)	0.8013 (3)	0.61555 (15)	0.0525 (7)	
C9	0.56167 (17)	0.7155 (3)	0.67046 (18)	0.0584 (8)	
H9	0.6058	0.6563	0.6658	0.070*	
C10	0.51327 (17)	0.7203 (3)	0.73259 (16)	0.0546 (7)	
H10	0.5262	0.6650	0.7709	0.066*	
C11	0.31097 (16)	0.6326 (2)	0.73959 (15)	0.0488 (7)	
C12	0.24278 (18)	0.6965 (3)	0.71096 (17)	0.0572 (7)	
H12	0.2159	0.7612	0.7375	0.069*	
C13	0.2139 (2)	0.6647 (3)	0.64262 (19)	0.0679 (9)	
H13	0.1672	0.7078	0.6235	0.082*	
C14	0.2530 (2)	0.5712 (3)	0.6032 (2)	0.0747 (10)	
H14	0.2336	0.5511	0.5569	0.090*	
C15	0.3214 (2)	0.5062 (3)	0.6317 (2)	0.0770 (10)	
H15	0.3484	0.4423	0.6045	0.092*	
C16	0.3502 (2)	0.5354 (3)	0.70058 (19)	0.0660 (8)	
H16	0.3955	0.4899	0.7204	0.079*	
N1	0.34542 (14)	0.6745 (2)	0.80829 (12)	0.0514 (6)	
N2	0.59028 (18)	0.7939 (3)	0.54789 (16)	0.0700 (7)	
O1	0.28131 (16)	0.5087 (2)	0.86495 (14)	0.0905 (8)	
O2	0.57444 (17)	0.8711 (3)	0.49997 (13)	0.0870 (8)	
O3	0.6441 (2)	0.7111 (3)	0.54203 (18)	0.1312 (13)	
S1	0.45206 (5)	0.83866 (9)	0.88301 (4)	0.0706 (3)	

### (2S)-2-(3-Nitrophenyl)-3-phenyl-3,4,5,6-tetrahydro-2H-1,3-thiazin-4-one (1) Atomic displacement parameters (Å^2^)

**Table:**

	*U*^11^	*U*^22^	*U*^33^	*U*^12^	*U*^13^	*U*^23^
C1	0.0452 (15)	0.0467 (14)	0.0561 (17)	0.0018 (11)	0.0019 (12)	0.0096 (11)
C2	0.0498 (16)	0.0657 (18)	0.068 (2)	0.0000 (14)	0.0077 (14)	0.0223 (16)
C3	0.065 (2)	0.099 (3)	0.060 (2)	0.0073 (18)	0.0096 (16)	0.0301 (18)
C4	0.072 (2)	0.100 (3)	0.0521 (19)	0.0153 (19)	0.0029 (16)	0.0062 (17)
C5	0.0395 (13)	0.0399 (12)	0.0543 (16)	−0.0048 (10)	−0.0009 (11)	0.0045 (11)
C6	0.0439 (14)	0.0458 (13)	0.0550 (17)	0.0024 (11)	−0.0026 (12)	0.0079 (12)
C7	0.0550 (16)	0.0586 (16)	0.0517 (17)	−0.0010 (14)	−0.0036 (13)	0.0106 (13)
C8	0.0445 (15)	0.0585 (16)	0.0545 (17)	−0.0061 (12)	0.0055 (13)	0.0029 (13)
C9	0.0436 (15)	0.0584 (16)	0.073 (2)	0.0060 (12)	0.0027 (14)	0.0090 (15)
C10	0.0481 (15)	0.0545 (15)	0.0613 (18)	0.0053 (13)	0.0041 (13)	0.0188 (13)
C11	0.0438 (14)	0.0466 (14)	0.0559 (17)	−0.0038 (11)	0.0047 (13)	0.0097 (12)
C12	0.0496 (16)	0.0574 (16)	0.065 (2)	0.0008 (13)	−0.0008 (14)	−0.0009 (14)
C13	0.0625 (19)	0.0674 (19)	0.074 (2)	−0.0120 (16)	−0.0130 (17)	0.0057 (17)
C14	0.083 (2)	0.073 (2)	0.069 (2)	−0.0263 (19)	−0.0012 (19)	−0.0034 (18)
C15	0.086 (3)	0.063 (2)	0.083 (3)	−0.0113 (18)	0.012 (2)	−0.0183 (18)
C16	0.0600 (18)	0.0513 (16)	0.087 (2)	0.0032 (14)	0.0041 (17)	0.0009 (15)
N1	0.0475 (13)	0.0509 (12)	0.0558 (15)	−0.0010 (10)	0.0032 (11)	0.0165 (10)
N2	0.0613 (16)	0.0829 (19)	0.0657 (19)	−0.0036 (15)	0.0121 (14)	0.0036 (15)
O1	0.0943 (18)	0.0839 (17)	0.0934 (19)	−0.0271 (14)	0.0132 (14)	0.0336 (14)
O2	0.0969 (18)	0.1087 (19)	0.0554 (15)	−0.0039 (15)	0.0103 (13)	0.0133 (14)
O3	0.124 (3)	0.155 (3)	0.115 (3)	0.062 (2)	0.061 (2)	0.030 (2)
S1	0.0682 (6)	0.0868 (6)	0.0569 (6)	−0.0123 (4)	−0.0040 (4)	0.0004 (4)

### (2S)-2-(3-Nitrophenyl)-3-phenyl-3,4,5,6-tetrahydro-2H-1,3-thiazin-4-one (1) Geometric parameters (Å, º)

**Table:**

C1—H1	0.9800	C8—C9	1.378 (4)
C1—C5	1.509 (4)	C8—N2	1.462 (4)
C1—N1	1.491 (3)	C9—H9	0.9300
C1—S1	1.795 (3)	C9—C10	1.379 (4)
C2—C3	1.495 (5)	C10—H10	0.9300
C2—N1	1.359 (3)	C11—C12	1.368 (4)
C2—O1	1.230 (4)	C11—C16	1.379 (4)
C3—H3A	0.9700	C11—N1	1.446 (4)
C3—H3B	0.9700	C12—H12	0.9300
C3—C4	1.503 (5)	C12—C13	1.381 (4)
C4—H4A	0.9700	C13—H13	0.9300
C4—H4B	0.9700	C13—C14	1.355 (5)
C4—S1	1.790 (3)	C14—H14	0.9300
C5—C6	1.378 (4)	C14—C15	1.375 (5)
C5—C10	1.388 (4)	C15—H15	0.9300
C6—H6	0.9300	C15—C16	1.384 (4)
C6—C7	1.379 (4)	C16—H16	0.9300
C7—H7	0.9300	N2—O2	1.215 (3)
C7—C8	1.375 (4)	N2—O3	1.208 (4)
			
C5—C1—H1	108.7	C9—C8—N2	118.7 (3)
C5—C1—S1	108.04 (18)	C8—C9—H9	121.1
N1—C1—H1	108.7	C8—C9—C10	117.8 (3)
N1—C1—C5	109.0 (2)	C10—C9—H9	121.1
N1—C1—S1	113.65 (17)	C5—C10—H10	119.3
S1—C1—H1	108.7	C9—C10—C5	121.4 (3)
N1—C2—C3	120.4 (3)	C9—C10—H10	119.3
O1—C2—C3	119.7 (3)	C12—C11—C16	120.0 (3)
O1—C2—N1	119.9 (3)	C12—C11—N1	119.5 (2)
C2—C3—H3A	108.0	C16—C11—N1	120.3 (3)
C2—C3—H3B	108.0	C11—C12—H12	120.0
C2—C3—C4	117.2 (2)	C11—C12—C13	120.0 (3)
H3A—C3—H3B	107.2	C13—C12—H12	120.0
C4—C3—H3A	108.0	C12—C13—H13	119.7
C4—C3—H3B	108.0	C14—C13—C12	120.5 (3)
C3—C4—H4A	109.7	C14—C13—H13	119.7
C3—C4—H4B	109.7	C13—C14—H14	120.1
C3—C4—S1	109.9 (2)	C13—C14—C15	119.8 (3)
H4A—C4—H4B	108.2	C15—C14—H14	120.1
S1—C4—H4A	109.7	C14—C15—H15	119.8
S1—C4—H4B	109.7	C14—C15—C16	120.3 (3)
C6—C5—C1	120.1 (2)	C16—C15—H15	119.8
C6—C5—C10	118.7 (2)	C11—C16—C15	119.3 (3)
C10—C5—C1	121.2 (2)	C11—C16—H16	120.3
C5—C6—H6	119.4	C15—C16—H16	120.3
C5—C6—C7	121.2 (2)	C2—N1—C1	126.3 (3)
C7—C6—H6	119.4	C2—N1—C11	118.8 (2)
C6—C7—H7	120.8	C11—N1—C1	113.49 (19)
C8—C7—C6	118.3 (3)	O2—N2—C8	118.8 (3)
C8—C7—H7	120.8	O3—N2—C8	118.5 (3)
C7—C8—C9	122.4 (3)	O3—N2—O2	122.7 (3)
C7—C8—N2	118.8 (3)	C4—S1—C1	95.08 (15)
			
C1—C5—C6—C7	−176.2 (2)	C12—C11—N1—C1	−69.7 (3)
C1—C5—C10—C9	175.0 (3)	C12—C11—N1—C2	97.7 (3)
C2—C3—C4—S1	−54.0 (4)	C12—C13—C14—C15	0.8 (5)
C3—C2—N1—C1	−5.9 (4)	C13—C14—C15—C16	0.3 (5)
C3—C2—N1—C11	−171.6 (3)	C14—C15—C16—C11	−1.7 (5)
C3—C4—S1—C1	63.8 (2)	C16—C11—C12—C13	−0.9 (4)
C5—C1—N1—C2	147.8 (3)	C16—C11—N1—C1	105.6 (3)
C5—C1—N1—C11	−45.9 (3)	C16—C11—N1—C2	−87.0 (3)
C5—C1—S1—C4	−171.67 (19)	N1—C1—C5—C6	115.7 (3)
C5—C6—C7—C8	0.4 (4)	N1—C1—C5—C10	−62.5 (3)
C6—C5—C10—C9	−3.3 (4)	N1—C1—S1—C4	−50.6 (2)
C6—C7—C8—C9	−1.9 (4)	N1—C2—C3—C4	20.1 (4)
C6—C7—C8—N2	176.0 (3)	N1—C11—C12—C13	174.4 (2)
C7—C8—C9—C10	0.7 (4)	N1—C11—C16—C15	−173.3 (3)
C7—C8—N2—O2	3.4 (4)	N2—C8—C9—C10	−177.1 (3)
C7—C8—N2—O3	−176.7 (3)	O1—C2—C3—C4	−160.7 (3)
C8—C9—C10—C5	1.9 (4)	O1—C2—N1—C1	174.9 (3)
C9—C8—N2—O2	−178.6 (3)	O1—C2—N1—C11	9.3 (4)
C9—C8—N2—O3	1.3 (4)	S1—C1—C5—C6	−120.4 (2)
C10—C5—C6—C7	2.1 (4)	S1—C1—C5—C10	61.4 (3)
C11—C12—C13—C14	−0.5 (5)	S1—C1—N1—C2	27.3 (3)
C12—C11—C16—C15	1.9 (4)	S1—C1—N1—C11	−166.44 (18)

### (2S)-2-(3-Nitrophenyl)-3-phenyl-3,4,5,6-tetrahydro-2H-1,3-thiazin-4-one (1) Hydrogen-bond geometry (Å, º)

**Table:**

*D*—H···*A*	*D*—H	H···*A*	*D*···*A*	*D*—H···*A*
C3—H3*A*···O2^i^	0.97	2.62	3.405 (4)	139
C3—H3*B*···O3^ii^	0.97	2.57	3.253 (5)	128
C7—H7···O2^iii^	0.93	2.50	3.417 (4)	170

Symmetry codes: (i) −*x*+1, *y*−1/2, −*z*+3/2; (ii) *x*−1/2, *y*, −*z*+3/2; (iii) −*x*+1, −*y*+2, −*z*+1.

### rac-2-(4-Nitrophenyl)-3-phenyl-3,4,5,6-tetrahydro-2H-1,3-thiazin-4-one (2) Crystal data

**Table:**

C_16_H_14_N_2_O_3_S	*D*_x_ = 1.373 Mg m^−^^3^
*M**_r_* = 314.35	Mo *K*α radiation, λ = 0.71073 Å
Orthorhombic, *P*2_1_2_1_2_1_	Cell parameters from 1695 reflections
*a* = 8.6877 (17) Å	θ = 2.6–27.4°
*b* = 9.6547 (19) Å	µ = 0.23 mm^−^^1^
*c* = 18.137 (4) Å	*T* = 298 K
*V* = 1521.3 (5) Å^3^	Block, colorless
*Z* = 4	0.21 × 0.19 × 0.18 mm
*F*(000) = 656	

### rac-2-(4-Nitrophenyl)-3-phenyl-3,4,5,6-tetrahydro-2H-1,3-thiazin-4-one (2) Data collection

**Table:**

Bruker SMART CCD area detector diffractometer	3775 independent reflections
Radiation source: fine-focus sealed tube	3144 reflections with *I* > 2σ(*I*)
Graphite monochromator	*R*_int_ = 0.035
Detector resolution: 8.34 pixels mm^-1^	θ_max_ = 28.3°, θ_min_ = 2.3°
phi and ω scans	*h* = −11→11
Absorption correction: multi-scan (SADABS; Bruker, 2001)	*k* = −12→12
*T*_min_ = 0.341, *T*_max_ = 0.9	*l* = −24→22
14176 measured reflections	

### rac-2-(4-Nitrophenyl)-3-phenyl-3,4,5,6-tetrahydro-2H-1,3-thiazin-4-one (2) Refinement

**Table:**

Refinement on *F*^2^	Secondary atom site location: difference Fourier map
Least-squares matrix: full	Hydrogen site location: inferred from neighbouring sites
*R*[*F*^2^ > 2σ(*F*^2^)] = 0.045	H-atom parameters constrained
*wR*(*F*^2^) = 0.121	*w* = 1/[σ^2^(*F*_o_^2^) + (0.0783*P*)^2^] where *P* = (*F*_o_^2^ + 2*F*_c_^2^)/3
*S* = 1.01	(Δ/σ)_max_ = 0.001
3775 reflections	Δρ_max_ = 0.32 e Å^−^^3^
199 parameters	Δρ_min_ = −0.16 e Å^−^^3^
0 restraints	Absolute structure: Flack (1983), 4055 Friedel pairs
Primary atom site location: structure-invariant direct methods	Absolute structure parameter: 0.09 (7)

### rac-2-(4-Nitrophenyl)-3-phenyl-3,4,5,6-tetrahydro-2H-1,3-thiazin-4-one (2) Special details

**Table:**

Experimental. The data collection nominally covered a full sphere of reciprocal space by a combination of 4 sets of ω scans each set at different φ and/or 2θ angles and each scan (10 s exposure) covering -0.300° degrees in ω. The crystal to detector distance was 5.82 cm.
Geometry. All esds (except the esd in the dihedral angle between two l.s. planes) are estimated using the full covariance matrix. The cell esds are taken into account individually in the estimation of esds in distances, angles and torsion angles; correlations between esds in cell parameters are only used when they are defined by crystal symmetry. An approximate (isotropic) treatment of cell esds is used for estimating esds involving l.s. planes.
Refinement. Refinement of F^2^ against ALL reflections. The weighted R-factor wR and goodness of fit S are based on F^2^, conventional R-factors R are based on F, with F set to zero for negative F^2^. The threshold expression of F^2^ > 2sigma(F^2^) is used only for calculating R-factors(gt) etc. and is not relevant to the choice of reflections for refinement. R-factors based on F^2^ are statistically about twice as large as those based on F, and R- factors based on ALL data will be even larger.

### rac-2-(4-Nitrophenyl)-3-phenyl-3,4,5,6-tetrahydro-2H-1,3-thiazin-4-one (2) Fractional atomic coordinates and isotropic or equivalent isotropic displacement parameters (Å^2^)

**Table:**

	*x*	*y*	*z*	*U*_iso_*/*U*_eq_	
C1	0.26199 (19)	0.53305 (17)	0.75480 (10)	0.0468 (4)	
H1	0.1885	0.6066	0.7426	0.056*	
C2	0.0844 (3)	0.3448 (2)	0.79727 (12)	0.0640 (5)	
C3	0.0753 (3)	0.4045 (3)	0.87435 (13)	0.0688 (6)	
H3A	−0.0321	0.4053	0.8889	0.083*	
H3B	0.1283	0.3413	0.9073	0.083*	
C4	0.1385 (2)	0.5458 (2)	0.88739 (13)	0.0685 (6)	
H4A	0.1435	0.5638	0.9400	0.082*	
H4B	0.0710	0.6143	0.8653	0.082*	
C5	0.39844 (19)	0.54561 (17)	0.70401 (10)	0.0462 (4)	
C6	0.3973 (2)	0.64968 (18)	0.65124 (11)	0.0499 (4)	
H6	0.3133	0.7089	0.6470	0.060*	
C7	0.5226 (2)	0.6638 (2)	0.60521 (11)	0.0535 (5)	
C8	0.6496 (2)	0.5792 (2)	0.60899 (13)	0.0614 (5)	
H8	0.7326	0.5913	0.5773	0.074*	
C9	0.6499 (2)	0.4757 (2)	0.66142 (14)	0.0648 (6)	
H9	0.7343	0.4168	0.6653	0.078*	
C10	0.5258 (2)	0.45870 (19)	0.70826 (12)	0.0546 (5)	
H10	0.5274	0.3881	0.7431	0.066*	
C11	0.17339 (19)	0.34283 (18)	0.67247 (10)	0.0464 (4)	
C12	0.0949 (2)	0.4149 (2)	0.61946 (11)	0.0541 (4)	
H12	0.0456	0.4973	0.6315	0.065*	
C13	0.0889 (3)	0.3649 (3)	0.54785 (13)	0.0697 (6)	
H13	0.0360	0.4136	0.5116	0.084*	
C14	0.1617 (3)	0.2433 (3)	0.53116 (13)	0.0784 (8)	
H14	0.1574	0.2088	0.4833	0.094*	
C15	0.2404 (3)	0.1724 (3)	0.58378 (16)	0.0748 (7)	
H15	0.2902	0.0903	0.5715	0.090*	
C16	0.2472 (2)	0.2211 (2)	0.65527 (13)	0.0601 (5)	
H16	0.3009	0.1723	0.6912	0.072*	
N1	0.18311 (17)	0.39713 (15)	0.74654 (8)	0.0473 (3)	
N2	0.5185 (3)	0.7744 (2)	0.54921 (11)	0.0695 (5)	
O1	0.0060 (2)	0.2427 (2)	0.78250 (10)	0.1044 (8)	
O2	0.6279 (3)	0.7859 (2)	0.50693 (12)	0.1032 (7)	
O3	0.4078 (2)	0.8498 (2)	0.54701 (13)	0.0971 (7)	
S1	0.32852 (6)	0.56010 (6)	0.84780 (3)	0.06274 (17)	

### rac-2-(4-Nitrophenyl)-3-phenyl-3,4,5,6-tetrahydro-2H-1,3-thiazin-4-one (2) Atomic displacement parameters (Å^2^)

**Table:**

	*U*^11^	*U*^22^	*U*^33^	*U*^12^	*U*^13^	*U*^23^
C1	0.0395 (8)	0.0424 (9)	0.0586 (10)	0.0006 (7)	−0.0046 (8)	0.0003 (8)
C2	0.0585 (11)	0.0712 (13)	0.0624 (12)	−0.0210 (11)	0.0031 (10)	−0.0021 (10)
C3	0.0573 (11)	0.0877 (16)	0.0613 (12)	−0.0131 (12)	0.0106 (9)	−0.0027 (11)
C4	0.0561 (11)	0.0838 (15)	0.0655 (12)	−0.0010 (11)	0.0092 (10)	−0.0206 (12)
C5	0.0399 (8)	0.0400 (8)	0.0587 (10)	−0.0030 (7)	−0.0068 (7)	−0.0026 (8)
C6	0.0473 (8)	0.0417 (9)	0.0606 (11)	−0.0030 (7)	−0.0123 (9)	−0.0026 (8)
C7	0.0648 (11)	0.0428 (9)	0.0529 (11)	−0.0117 (9)	−0.0069 (9)	−0.0005 (8)
C8	0.0599 (11)	0.0527 (11)	0.0717 (13)	−0.0094 (10)	0.0122 (10)	−0.0040 (10)
C9	0.0482 (9)	0.0530 (11)	0.0932 (16)	0.0051 (8)	0.0087 (11)	0.0020 (11)
C10	0.0489 (9)	0.0449 (10)	0.0701 (12)	0.0015 (8)	−0.0016 (9)	0.0080 (9)
C11	0.0377 (7)	0.0471 (9)	0.0544 (9)	−0.0070 (7)	−0.0012 (8)	−0.0017 (7)
C12	0.0478 (9)	0.0525 (10)	0.0621 (11)	−0.0027 (8)	−0.0081 (9)	0.0016 (9)
C13	0.0786 (14)	0.0757 (15)	0.0547 (12)	−0.0185 (13)	−0.0091 (12)	0.0099 (11)
C14	0.0955 (18)	0.0839 (17)	0.0556 (12)	−0.0344 (16)	0.0165 (13)	−0.0110 (12)
C15	0.0721 (14)	0.0633 (13)	0.0891 (17)	−0.0018 (12)	0.0275 (13)	−0.0183 (13)
C16	0.0514 (10)	0.0550 (11)	0.0741 (13)	0.0058 (9)	−0.0007 (10)	0.0007 (11)
N1	0.0431 (7)	0.0500 (8)	0.0489 (8)	−0.0088 (6)	−0.0029 (6)	−0.0002 (7)
N2	0.0846 (14)	0.0604 (11)	0.0637 (11)	−0.0208 (11)	−0.0131 (10)	0.0075 (9)
O1	0.1126 (16)	0.1198 (15)	0.0807 (11)	−0.0768 (14)	0.0227 (11)	−0.0220 (11)
O2	0.133 (2)	0.0990 (14)	0.0779 (11)	−0.0127 (13)	0.0272 (13)	0.0221 (11)
O3	0.0867 (12)	0.0827 (13)	0.1218 (16)	−0.0107 (11)	−0.0210 (12)	0.0455 (12)
S1	0.0486 (2)	0.0780 (4)	0.0616 (3)	−0.0121 (2)	−0.0044 (2)	−0.0156 (3)

### rac-2-(4-Nitrophenyl)-3-phenyl-3,4,5,6-tetrahydro-2H-1,3-thiazin-4-one (2) Geometric parameters (Å, º)

**Table:**

C1—H1	0.9800	C8—H8	0.9300
C1—C5	1.506 (2)	C8—C9	1.380 (3)
C1—N1	1.488 (2)	C9—H9	0.9300
C1—S1	1.802 (2)	C9—C10	1.383 (3)
C2—C3	1.514 (3)	C10—H10	0.9300
C2—N1	1.356 (3)	C11—C12	1.369 (3)
C2—O1	1.228 (3)	C11—C16	1.375 (3)
C3—H3A	0.9700	C11—N1	1.444 (2)
C3—H3B	0.9700	C12—H12	0.9300
C3—C4	1.490 (3)	C12—C13	1.386 (3)
C4—H4A	0.9700	C13—H13	0.9300
C4—H4B	0.9700	C13—C14	1.368 (4)
C4—S1	1.806 (2)	C14—H14	0.9300
C5—C6	1.388 (3)	C14—C15	1.359 (4)
C5—C10	1.391 (2)	C15—H15	0.9300
C6—H6	0.9300	C15—C16	1.380 (4)
C6—C7	1.379 (3)	C16—H16	0.9300
C7—C8	1.375 (3)	N2—O2	1.226 (3)
C7—N2	1.474 (3)	N2—O3	1.207 (3)
			
C5—C1—H1	108.4	C9—C8—H8	121.1
C5—C1—S1	107.96 (11)	C8—C9—H9	119.7
N1—C1—H1	108.4	C8—C9—C10	120.51 (19)
N1—C1—C5	111.83 (14)	C10—C9—H9	119.7
N1—C1—S1	111.70 (12)	C5—C10—H10	119.5
S1—C1—H1	108.4	C9—C10—C5	121.00 (18)
N1—C2—C3	121.17 (18)	C9—C10—H10	119.5
O1—C2—C3	118.6 (2)	C12—C11—C16	120.49 (19)
O1—C2—N1	120.1 (2)	C12—C11—N1	119.87 (16)
C2—C3—H3A	107.7	C16—C11—N1	119.61 (18)
C2—C3—H3B	107.7	C11—C12—H12	120.0
H3A—C3—H3B	107.1	C11—C12—C13	120.0 (2)
C4—C3—C2	118.4 (2)	C13—C12—H12	120.0
C4—C3—H3A	107.7	C12—C13—H13	120.4
C4—C3—H3B	107.7	C14—C13—C12	119.2 (2)
C3—C4—H4A	109.6	C14—C13—H13	120.4
C3—C4—H4B	109.6	C13—C14—H14	119.7
C3—C4—S1	110.09 (15)	C15—C14—C13	120.6 (2)
H4A—C4—H4B	108.2	C15—C14—H14	119.7
S1—C4—H4A	109.6	C14—C15—H15	119.7
S1—C4—H4B	109.6	C14—C15—C16	120.6 (2)
C6—C5—C1	118.31 (16)	C16—C15—H15	119.7
C6—C5—C10	118.75 (17)	C11—C16—C15	119.0 (2)
C10—C5—C1	122.93 (16)	C11—C16—H16	120.5
C5—C6—H6	120.5	C15—C16—H16	120.5
C7—C6—C5	118.90 (17)	C2—N1—C1	123.49 (16)
C7—C6—H6	120.5	C2—N1—C11	117.31 (15)
C6—C7—N2	118.02 (19)	C11—N1—C1	116.14 (14)
C8—C7—C6	123.01 (18)	O2—N2—C7	118.5 (2)
C8—C7—N2	119.0 (2)	O3—N2—C7	118.6 (2)
C7—C8—H8	121.1	O3—N2—O2	122.8 (2)
C7—C8—C9	117.81 (19)	C1—S1—C4	93.89 (9)
			
C1—C5—C6—C7	178.90 (16)	C12—C11—N1—C1	−61.8 (2)
C1—C5—C10—C9	−178.72 (19)	C12—C11—N1—C2	99.1 (2)
C2—C3—C4—S1	−48.3 (3)	C12—C13—C14—C15	−0.6 (4)
C3—C2—N1—C1	−14.7 (3)	C13—C14—C15—C16	0.6 (4)
C3—C2—N1—C11	−174.1 (2)	C14—C15—C16—C11	−0.2 (4)
C3—C4—S1—C1	63.17 (18)	C16—C11—C12—C13	0.3 (3)
C5—C1—N1—C2	162.42 (18)	C16—C11—N1—C1	116.17 (18)
C5—C1—N1—C11	−37.9 (2)	C16—C11—N1—C2	−82.9 (2)
C5—C1—S1—C4	177.36 (13)	N1—C1—C5—C6	118.45 (17)
C5—C6—C7—C8	0.1 (3)	N1—C1—C5—C10	−62.3 (2)
C5—C6—C7—N2	179.58 (16)	N1—C1—S1—C4	−59.28 (14)
C6—C5—C10—C9	0.5 (3)	N1—C2—C3—C4	18.6 (4)
C6—C7—C8—C9	0.1 (3)	N1—C11—C12—C13	178.24 (18)
C6—C7—N2—O2	−178.4 (2)	N1—C11—C16—C15	−178.26 (19)
C6—C7—N2—O3	1.7 (3)	N2—C7—C8—C9	−179.38 (18)
C7—C8—C9—C10	0.0 (3)	O1—C2—C3—C4	−165.2 (2)
C8—C7—N2—O2	1.1 (3)	O1—C2—N1—C1	169.2 (2)
C8—C7—N2—O3	−178.8 (2)	O1—C2—N1—C11	9.8 (3)
C8—C9—C10—C5	−0.4 (3)	S1—C1—C5—C6	−118.27 (14)
C10—C5—C6—C7	−0.4 (3)	S1—C1—C5—C10	61.0 (2)
C11—C12—C13—C14	0.2 (3)	S1—C1—N1—C2	41.3 (2)
C12—C11—C16—C15	−0.3 (3)	S1—C1—N1—C11	−159.05 (12)

### rac-2-(4-Nitrophenyl)-3-phenyl-3,4,5,6-tetrahydro-2H-1,3-thiazin-4-one (2) Hydrogen-bond geometry (Å, º)

**Table:**

*D*—H···*A*	*D*—H	H···*A*	*D*···*A*	*D*—H···*A*
C1—H1···O1^i^	0.98	2.19	3.158 (2)	170
C15—H15···O3^ii^	0.93	2.58	3.501 (3)	174

Symmetry codes: (i) −*x*, *y*+1/2, −*z*+3/2; (ii) *x*, *y*−1, *z*.

## References

[bb1] Arya, K., Rawat, D. S., Dandia, A. & Sasai, H. M. (2012). *J. Fluor. Chem.* **137**, 117–122.

[bb2] Bourhis, L. J., Dolomanov, O. V., Gildea, R. J., Howard, J. A. K. & Puschmann, H. (2015). *Acta Cryst.* A**71**, 59–75. 10.1107/S2053273314022207 PMC4283469 25537389

[bb3] Bredikhin, A. A. & Bredikhina, Z. A. (2017). *Chem. Eng. Technol.* **40**, 1211–1220.

[bb21] Bruker (2001). *SMART*, *SAINT* and *SADABS*. Bruker AXS Inc., Madison, Wisconsin, USA.

[bb4] Chen, Y., Wu, J. Yu. L., Zhai, D., Yi, Z., Luo, J. N. & Liu, M. (2012). Patent CN102653526A.

[bb5] Choi, H., Wang, Z., Zhu, X., He, X., Yang, K. & Liu, H. (2008). Patent WO2008112674, A1.

[bb6] Dandia, A., Singh, R. & Arya, K. (2004). *Phosphorus Sulfur Silicon*, **179**, 551–564.

[bb7] Dandia, A., Singh, R. & Saini, D. (2013). *J. Chem. Sci.* **125**, 1045–1053.

[bb8] Dolomanov, O. V., Bourhis, L. J., Gildea, R. J., Howard, J. A. K. & Puschmann, H. (2009). *J. Appl. Cryst.* **42**, 339–341.

[bb9] D’Oria, E., Karamertzanis, P. G. & Price, S. L. (2010). *Cryst. Growth Des.* **10**, 1749–1756.

[bb10] Eliel, E. & Wilen, S. H. (1994). *Stereochemistry of Organic Compounds.* New York: John Wiley & Sons.

[bb11] Flack, H. D. (1983). *Acta Cryst.* A**39**, 876–881.

[bb12] Jacques, J., Collet, A. & Wilen, S. H. (1981). *Enantiomers, Racemates, and Resolutions.* New York: John Wiley & Sons.

[bb13] Krumkains, E. V. (1984). *EP*, **10420**, B1.

[bb14] Pasteur, L. (1848). *Ann. Chim. Phys.* **22**, 442–459.

[bb15] Pérez-García, L. & Amabilino, D. B. (2007). *Chem. Soc. Rev.* **36**, 941–967. 10.1039/b610714a 17534480

[bb16] Qu, H., Zhang, R., Hu, Y., Ke, Y., Gao, Z. & Xu, H. (2013). *J. Biosci.* **68**, 77–81. 23819301

[bb17] Sheldrick, G. M. (2008). *Acta Cryst.* A**64**, 112–122. 10.1107/S0108767307043930 18156677

[bb18] Yennawar, H., Cali, A. S., Xie, Y. & Silverberg, L. J. (2015). *Acta Cryst.* E**71**, 414–417. 10.1107/S2056989015004545 PMC4438845 26029403

[bb19] Yennawar, H. P. & Silverberg, L. J. (2014). *Acta Cryst.* E**70**, o133. Corrigendum: (2015), E**71**, e5.

[bb22] Yennawar, H. P. & Silverberg, L. J. (2015), E**71**, e5. 10.1107/S2056989015022963 PMC4719803 26868441

[bb20] Yennawar, H. P., Silverberg, L. J., Minehan, M. J. & Tierney, J. (2013). *Acta Cryst.* E**69**, o1679. 10.1107/S1600536813028389 PMC3884335 24454111

